# Deficiency of apoptosis-stimulating protein two of p53 promotes liver regeneration in mice by activating mammalian target of rapamycin

**DOI:** 10.1038/s41598-018-36208-3

**Published:** 2018-12-18

**Authors:** Hongbo Shi, Yizhi Zhang, Jing Ji, Ping Xu, Honglin Shi, Xiujuan Yue, Feng Ren, Yu Chen, Zhongping Duan, Dexi Chen

**Affiliations:** 1 Beijing Youan Hospital, Capital Medical University, Beijing, 100069 China; 2 Beijing Institute of Hepatology, Capital Medical University, Beijing, 100069 China; 3 Beijing Precision Medicine and Transformation Engineering Technology Research Center of Hepatitis and Liver Center, Beijing, 100069 China; 4 Shanxi Provincial People’s Hospital Affiliated to Shanxi Medical University, Shanxi, China

## Abstract

Although liver regeneration has been intensively studied in various ways, the mechanisms underlying liver regeneration remain elusive. Apoptosis-stimulating protein two of p53 (ASPP2) was discovered as a binding partner of p53 and plays an important role in regulating cell apoptosis and growth. However, the role of ASPP2 in hepatocyte proliferation and liver regeneration has not been reported. The expression profile of ASPP2 was measured in a mouse model with 70% partial hepatectomy (PH_X_). Liver regeneration and hepatocyte proliferation were detected in wild-type (ASPP2^+/+^) and ASPP2 haploinsufficient (ASPP2^+/−^) mice with PH_X_. The mammalian target of rapamycin (mTOR) and autophagy pathways were analyzed in the ASPP2^+/+^ and ASPP2^+/−^ mice with PH_X_. After rapamycin or 3-methyladenine (3-MA) treatment, hepatocyte proliferation and liver regeneration were analyzed in the ASPP2^+/+^ and ASPP2^+/−^ mice with PH_X_. ASPP2 expression was shown to be upregulated at the early stage and downregulated at the late stage. Compared to the ASPP2^+/+^ mice, liver regeneration was enhanced in ASPP2^+/−^ mice with 70% PH_X_. In addition, compared to the ASPP2^+/+^ mice, the mTORC1 pathway was significantly upregulated and the autophagic pathway was downregulated in ASPP2^+/−^mice with 70% PH_X_. Inhibition of the mTORC1 pathway significantly suppressed liver regeneration in ASPP2^+/−^ mice with 70% PH_X_. In contrast, disruption of the autophagic pathway further enhanced liver regeneration in ASPP2^+/−^ mice with 70% PH_X_. ASPP2 deficiency can promote liver regeneration through activating the mTORC1 pathway, which further regulates downstream molecules, such as those related to autophagy and p70S6K expression in mouse model post-PH_X_.

## Introduction

After a partial hepatectomy (PH) or liver injury, hepatocytes and the other nonparenchymal hepatocytes in G0 phage enter into the cell cycle rapidly, and the remaining liver restore the original mass, structure and function, which is a process called liver regeneration^1^. Liver regeneration is regulated by many cytokines and growth factors, such as tumor necrosis factor α (TNF-α), interleukin six (IL-6), hepatocyte growth factor (HGF), transforming growth factor α (TGF-α), transforming growth factor β (TGF-β) and others. TNF-α and IL-6 act as the initial factors in liver regeneration, HGF, TGF-α and cyclin proteins play important roles in cell cycle entry and proliferation, and TGF-β is involved in the cessation of cell division^2–4^. Although liver regeneration has been intensively studied in various ways, the underlying mechanisms remain elusive.

Apoptosis-stimulating protein 2 of p53 (ASPP2) is a member of the ASPP family, which regulatesp53-dependent apoptosis, specifically^5^. ASPP2, which was discovered as a binding partner of p53 more than 10 years ago, plays an important role in regulating cell apoptosis and growth. In the case of stress or DNA damage, ASPP2 is activated and induces cell apoptosis. On the other hand, methylation of the ASPP2 promoter results in ASPP2 inactivation and cell growth^6,7^. Studies on ASPP2 have primarily focused on cancer, as ASPP2 expression is downregulated in many human tumors^8–10^. In liver cancer, the overexpression of ASPP2 promotes tumor cell apoptosis, and a deficiency of ASPP2 causes tumor cell growth and proliferation^9,10^. Apart from liver cancer, there are a few reports on ASPP2 in liver injury. Xie *et al*. found that ASPP2 overexpression suppressed methionine and choline-deficient (MCD) diet-induced autophagy, steatosis and apoptosis and reduced liver injury in a non-alcoholic fatty liver disease (NAFLD) mouse model, which indicated that ASPP2 may participate in hepatocyte apoptosis and liver injury^11^. However, the role of ASPP2 in liver regeneration and hepatocyte proliferation has not been reported.

Given the role of ASPP2 in cell apoptosis and growth, we speculated that the deletion of ASPP2 would promote liver regeneration and hepatocyte proliferation. The results of this study were consistent with our hypothesis. In addition, we found that enhanced liver regeneration in ASPP2 haploinsufficient (ASPP2^+/−^) mice after PH_X_ was dependent on the mammalian target of rapamycin (mTOR) pathway.

## Materials and Methods

### Animals

ASPP2^+/+^ Balb/c mice (aged 6 to 8 weeks) were provided by the Animal Center at the Academy of Military Medical Sciences (Beijing, China). ASPP2^+/−^ Balb/c mice (aged 6 to 8 weeks) were provided by the Animal Center of Beijing Institute of Hepatology (Beijing, China). All experimental protocols were approved by the Ethics Committee of Beijing Youan Hospital. All methods were performed in accordance with relevant guidelines and regulations.

### PH_X_ Experiments

The mice were anesthetized with chloral hydrate (4 g/100 mL) and subjected to approximately 70% PHx by removing the left, median and posterior right lobes after a midventral laparotomy. The control group was anesthetized and subjected to an abdominal incision, but no liver lobes were removed. The mice were scarified at 6 h, 12 h, 24 h, 48 h and 72 h after the PHx. The inhibition of mTORC1 was induced by an intraperitoneal injection of rapamycin (2.5 mg/kg, Sigma) 2 h before the PHx. The inhibition of autophagy was induced by a tail vein injection of 3-methyladenine (3-MA, 1 mg/kg, Sigma) 2 h before the PHx.

### Immunohistochemical analysis

The liver samples were fixed in buffered formalin, embedded in paraffin and cut into 5-um sections. The sections were deparaffinized, washed in PBS and blocked with normal goat serum. Then, the sections were incubated with a rabbit monoclonal antibody against proliferative cell nuclear antigen (PCNA) (Cell Signaling, CA, USA) overnight. After that, the sections were incubated with a goat anti-rabbit secondary antibody conjugated with horseradish peroxidase (Cell Signaling, CA, USA) for 30 minutes at 37 °C. After being washed with PBS, the sections were stained with diaminobenzidine substrate solution. All images were collected using a light microscope (Nikon Eclipse E800, Tokyo, Japan).

### Western blotting analysis

Liver tissues were lysed with a homogenizer in RIPA buffer with protease inhibitors. Total proteins were extracted using centrifugation and denatured via boiling. Then, 50 μg of protein were loaded onto a 12% separation gel and a 5% concentration gel. The total proteins were separated into many bands by electrophoresis and subsequently transferred to polyvinylidene difluoride (PVDF) membranes (Bio-Rad, CA, USA) by electroblotting. The membranes were incubated with the rabbit primary antibodies against ASPP2, LC3B (Sigma, MO, USA), p62, Atg7, Beclin-1, CyclinA2, CyclinB1, CyclinE1, phospho-4EBP1 T37/46, phospho-S6 S235/236, and phospho-P70S6K T389 (Cell Signaling, CA, USA). Then, the membranes were incubated with a goat anti-rabbit secondary antibody conjugated with horseradish peroxidase (Cell Signaling, CA, USA). Finally, immunoreactive bands were developed using a chemiluminescent substrate (Thermo Fisher Scientific, IL, USA). The grayscale was analyzed by ImageJ software, and the relative grayscale value was normalized to that of β-actin.

### Statistical analysis

Variables among different groups were compared by One-Way ANOVA for the post hoc multiple comparisons (LSD method) using the SPSS software package. Differences were considered significant when the p value was less than 0.05.

## Results

### ASPP2 expression profile during liver regeneration in mice after 70% PH_X_

**Figure 1:**
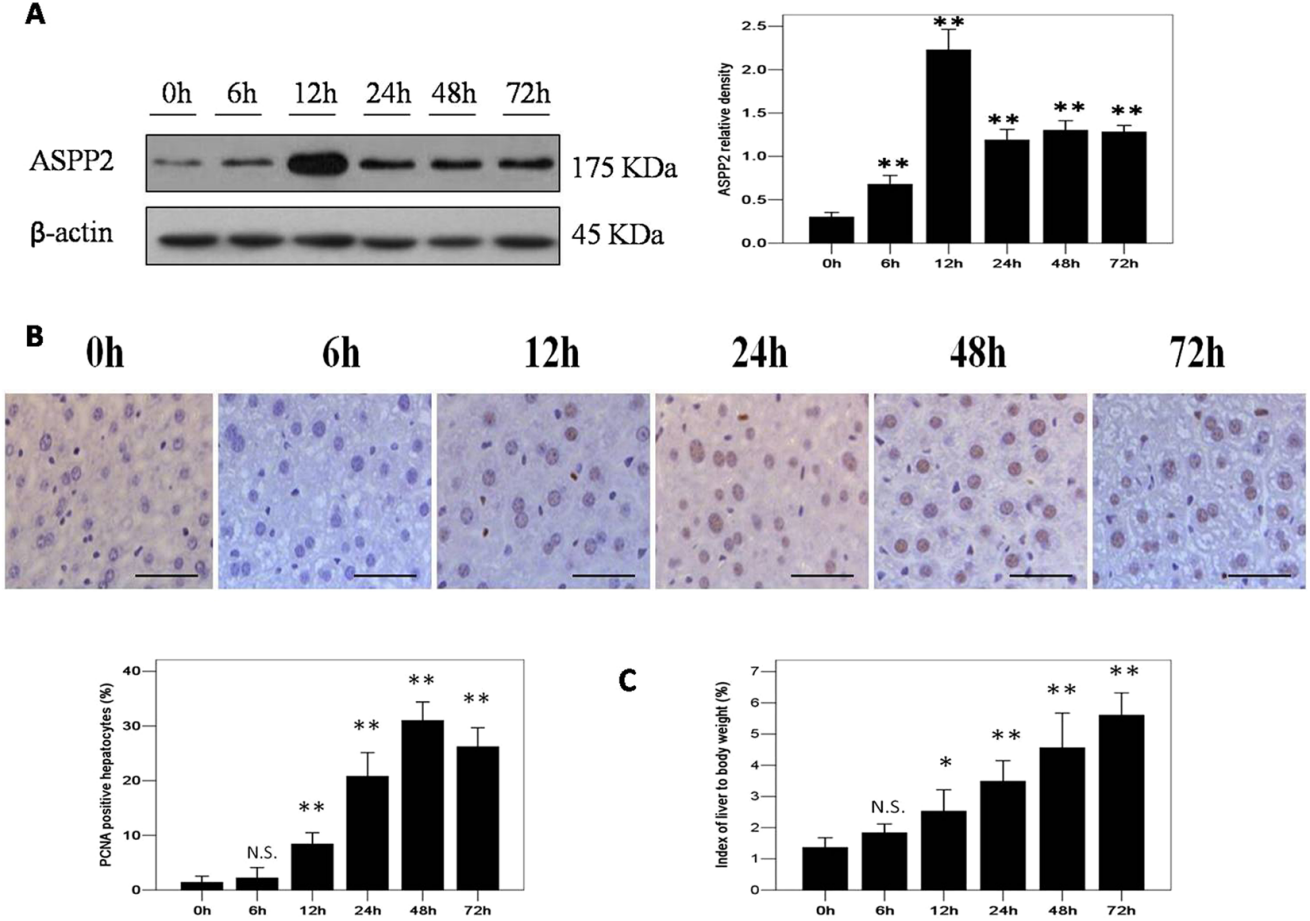
ASPP2 expression profile during liver regeneration in mice after 70% PH_X_. (**A**) Representative western blotting analysis of ASPP2 protein in the livers of ASPP2^+/+^ mice at 0 h, 6 h, 12 h, 24 h, 48 h, and 72 h after 70% PH_X_. Quantifications were normalized to β-actin (n = 5 for each time point). (**B**) Representative IHC staining of PCNA-positive hepatocytes in the livers of ASPP2^+/+^ mice at 0 h, 6 h, 12 h, 24 h, 48 h, and 72 h after 70% PH_X_. Hepatocyte nuclei were counted in 10 microscopic vision fields per section. Three sections per mouse were examined (n = 5 for each time point). Scale bar: 100 μm. (**C**) Index of liver to body weight in ASPP2^+/+^ mice at 0 h, 6 h, 12 h, 24 h, 48 h, and 72 h after 70% PH_X_ (n = 10 for each time point). Error bars represent the mean ± standard deviation (SD). *P < 0.05; *P < 0.01. Abbreviations: h, hour; N.S., no significant difference.

To investigate the role of ASPP2 in liver regeneration, we examined expression profile of ASPP2 in wild-type mice after 70% PH_X_. The number of proliferative cell nuclear antigen (PCNA)-positive hepatocytes was significantly increased in mice after 70% PH_X_ (Fig. 1B). In addition, the index of liver to body weight was significantly increased in mice after 70% PH_X_ (Fig. 1C). Accompanied with liver regeneration, the ASPP2 protein displayed a significant upregulation at the early stage (12 hours after PH_X_) and a significant downregulation at the late stage (24, 48 and 72 hours after PH_X_) (Fig. 1A). These results indicate that ASPP2 may play an important role in liver regeneration.

### Enhanced liver regeneration in ASPP2^+/−^ mice after 70% PH_X_

**Figure 2:**
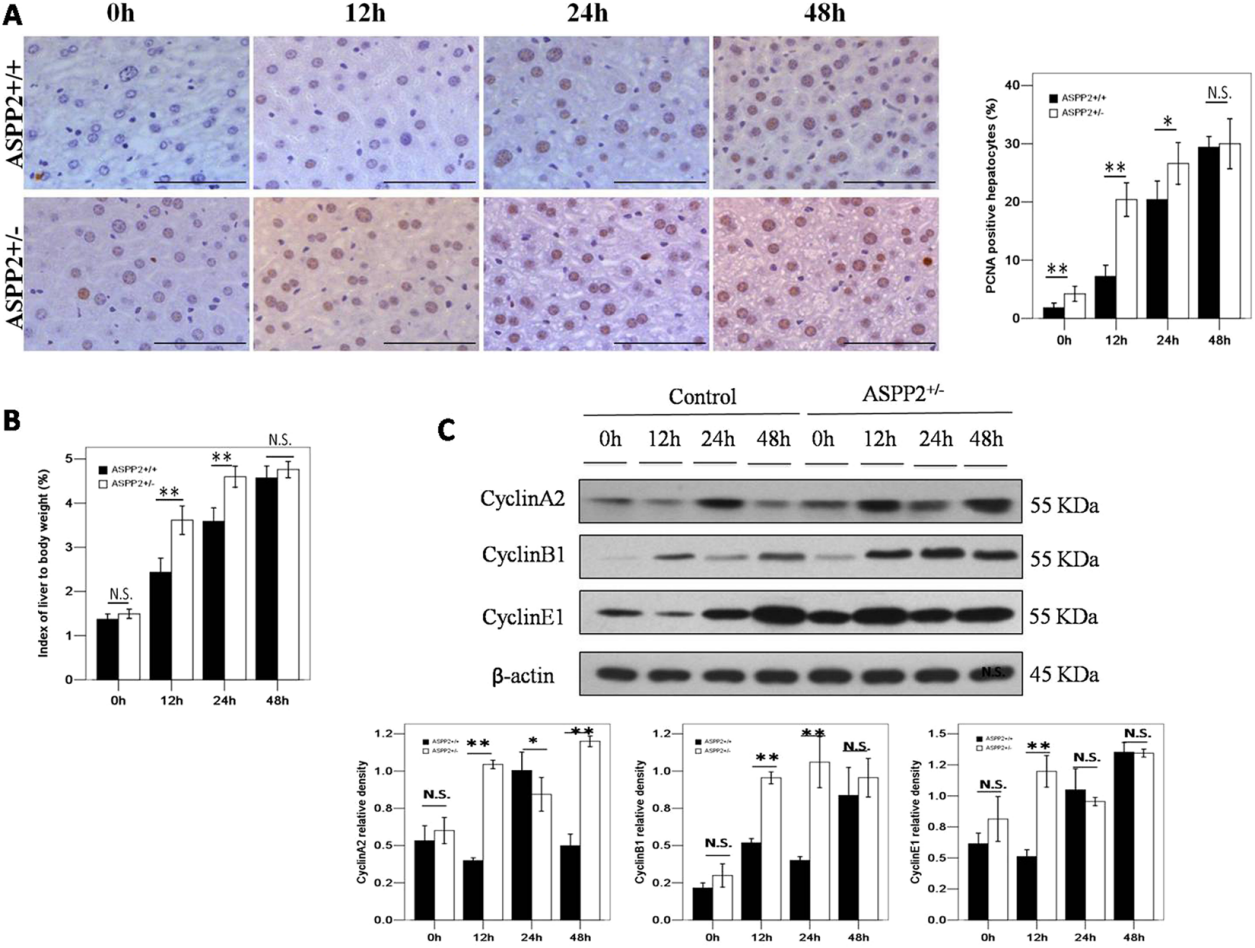
Enhanced liver regeneration in ASPP2^+/−^ mice after 70% PH_X_. (**A**) Representative IHC staining of PCNA-positive hepatocytes in the livers of ASPP2^+/+^ and ASPP2^+/−^ mice at 0 h, 12 h, 24 h, and 48 h after 70% PH_X_. Hepatocyte nuclei were counted in 10 microscopic vision fields per section. Three sections per mouse were examined (n = 5 for each time point). Scale bar: 100 μm. (**B**) Index of liver to body weight in ASPP2^+/+^ and ASPP2^+/−^ mice at 0 h, 12 h, 24 h, and 48 h after 70% PH_X_ (n = 10 for each time point). (**C**) Representative western blotting analysis of CyclinA2, CyclinB1 and CyclinD1 expression in the livers of ASPP2^+/+^ and ASPP2^+/−^ mice at 0 h, 12 h, 24 h, and 48 h after 70% PH_X_. Quantifications were normalized to β-actin (n = 5 for each time point). Error bars represent the mean ± standard deviation (SD). *P < 0.05; *P < 0.01. Abbreviations: h, hour; N.S., no significant difference.

To further investigate the role of ASPP2 in liver regeneration, we disrupted the gene expression of ASPP2 and examined liver regeneration in ASPP2^+/−^ mice after 70% PH_X_. It is known that cyclins are evolutionarily conserved proteins that are essential for cell-cycle control in eukaryotes^12^. The expression levels of cyclinA2, cyclinB1 and cyclinE1 were significantly higher in ASPP2^+/−^ mice than in ASPP2^+/+^ mice at 12 hours after PH_X_ (Fig. 2C). In addition, the index of liver to body weight in ASPP2^+/−^ mice was significantly increased in comparison to the ASPP2^+/+^ mice at 12 and 24 hours after PH_X_ (Fig. 2B). Consistently, the number of PCNA-positive hepatocytes in ASPP2^+/−^ mice were significantly increased compared to the ASPP2^+/+^ mice at 12 and 24 hours after PH_X_ (Fig. 2A). Taken together, these data indicate that liver regeneration was enhanced in the ASPP2^+/−^ mice after 70% PH_X_.

### Deficiency of ASPP2 activates the mTORC1 pathway in mice after 70% PH_X_

**Figure 3:**
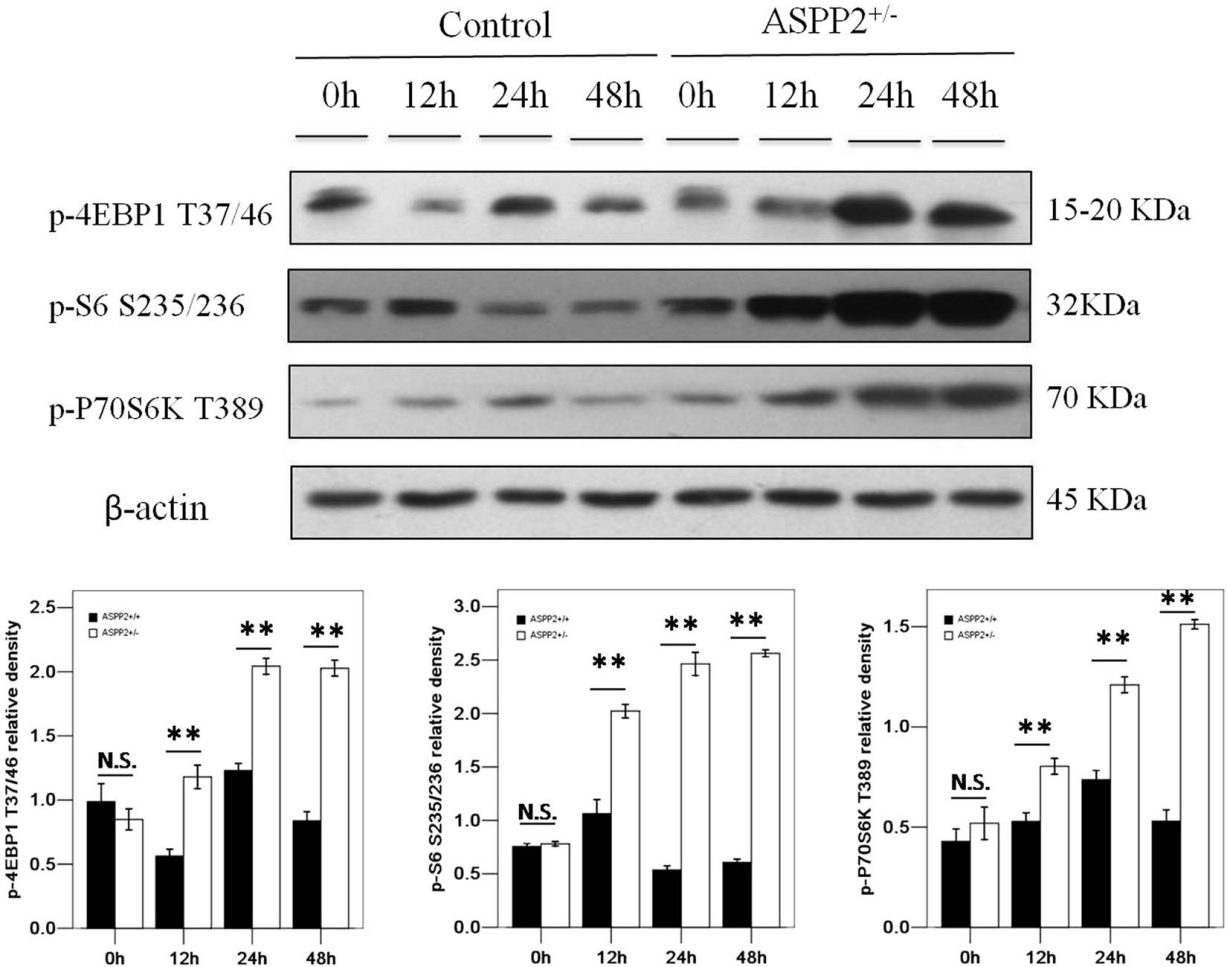
Inhibition of ASPP2 activates the mTORC1 pathway in mice with 70% PH_X_. Representative western blotting analysis of phospho-4EBP1 (Thr37/46), phospho-S6 (Ser235/236), phospho-P70S6K (Thr389) in the livers of ASPP2^+/+^ and ASPP2^+/−^ mice at 0 h, 12 h, 24 h, and 48 h after 70% PH_X_. Quantifications were normalized to β-actin (n = 5 for each time point). Error bars represent the mean ± standard deviation (SD). *P < 0.05; *P < 0.01. Abbreviations: h, hour; N.S., no significant difference.

Considering that the mTOR complex 1 (mTORC1) pathway is the primary pathway that regulates liver regeneration, we examined mTORC1 activity in ASPP2^+/−^ mice after 70% PH_X_. Compared to the ASPP2^+/+^ mice, the ASPP2^+/−^ mice exhibited significantly increased levels of phospho-4EBP1 (Thr37/46), phospho-S6 (Ser235/236) and phospho-P70S6K (Thr389) at 12, 24 and 48 hours after PH_X_ (Fig. 3). These findings demonstrated that a deficiency of ASPP2 activated the mTORC1 pathway, indicating that the mTORC1 pathway was likely responsible for enhanced liver regeneration in the ASPP2^+/−^ mice after 70% PH_X_.

### Deficiency of ASPP2 suppresses the autophagic pathway in mice after 70% PH_X_

**Figure 4:**
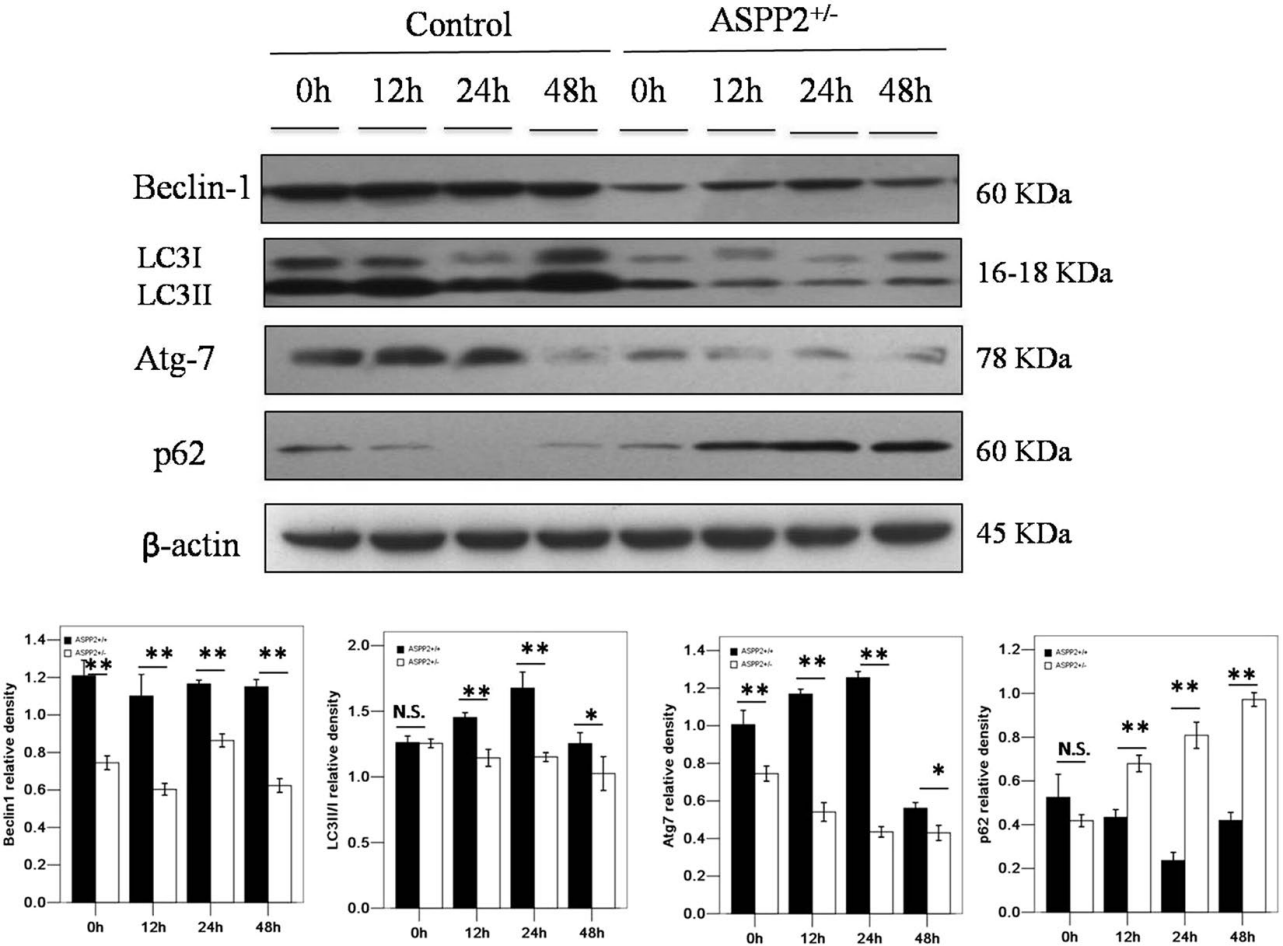
Inhibition of ASPP2 suppresses the autophagic pathway in mice with 70% PH_X_. Representative western blotting analysis of Beclin-1, LC3, Atg-7 and p62 in the livers of ASPP2^+/+^ and ASPP2^+/−^ mice at 0 h, 12 h, 24 h, and 48 h after 70% PH_X_. Quantifications were normalized to β-actin (n = 5 for each time point). Error bars represent the mean ± standard deviation (SD). *P < 0.05; *P < 0.01. Abbreviations: h, hour; N.S., no significant difference.

Previous studies indicated that ASPP2 regulates the autophagic pathway, which plays an important role in liver regeneration; therefore, we analyzed the role of the autophagic pathway in ASPP2^+/−^ mice after 70% PH_X_. The conversion of LC3II and degradation of p62 were significantly lower in the ASPP2^+/−^ mice than in ASPP2^+/+^ mice after PH_X_. Consistently, Beclin-1 and Atg-7 expression were decreased in the ASPP2^+/−^ mice compared with the ASPP2^+/+^ mice after PH_X_ (Fig. 4). These data showed that the knockdown of ASPP2 suppressed the autophagic pathway, which may participate in the regulation of liver regeneration through ASPP2.

### Inhibition of the mTORC1 pathway significantly suppresses liver regeneration in ASPP2^+/−^ mice

**Figure 5:**
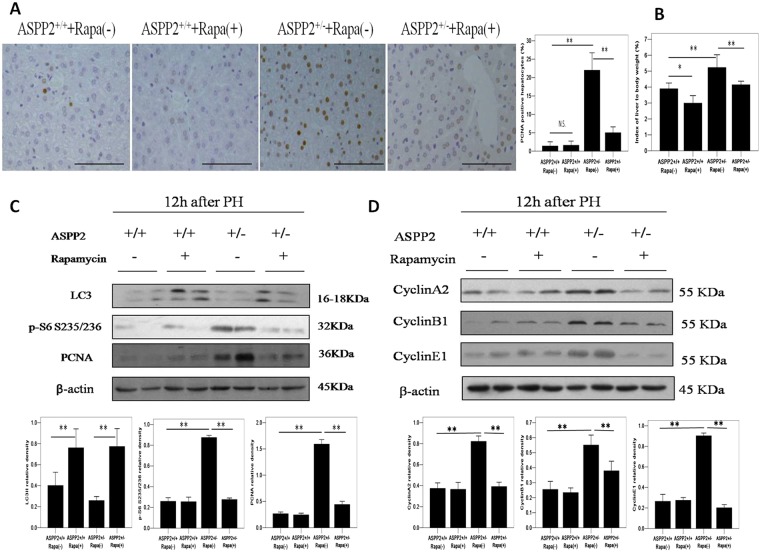
Inhibition of the mTORC1 pathway significantly suppresses liver regeneration in ASPP2^+/−^ mice. (**A**) Representative IHC staining of PCNA-positive hepatocytes in the livers of ASPP2^+/+^ and ASPP2^+/−^ mice treated with rapamycin or vehicle at 12 h after 70% PHx. Hepatocyte nuclei were counted in 10 microscopic vision fields per section. Three sections per mouse were examined (n = 5 for each time point). Scale bar: 100 μm. (**B**) Index of liver to body weight in ASPP2^+/+^ and ASPP2^+/−^ mice treated with rapamycin or vehicle at 24 h after 70% PHx (n = 5 for each time point). (**C**) Representative western blotting analysis of PCNA, LC3 and phospho-S6 (Ser235/236) expression in the livers of ASPP2^+/+^ and ASPP2^+/−^ mice treated with rapamycin or vehicle at 12 h after 70% PHx. (**D**) Representative western blotting analysis of CyclinA2, CyclinB1 and CyclinE1 expression in the livers of ASPP2^+/+^ and ASPP2^+/−^ mice treated with rapamycin or vehicle at 12 h after 70% PHx. Quantifications were normalized to β-actin (n = 5 for each time point). Error bars represent the mean ± standard deviation (SD). *P < 0.05; *P < 0.01. Abbreviations: h, hour; N.S., no significant difference.

To investigate the role of the mTORC1 pathway in the ASPP2-mediated regulation of liver regeneration, we used rapamycin to block the mTORC1 pathway in ASPP2^+/+^ and ASPP2^+/−^ mice after 70% PH_X_. As expected, rapamycin treatment inhibited the expression of phospho-S6 (Ser235/236) in ASPP2^+/+^ and ASPP2^+/−^ mice (Fig. 5C). Remarkably, the expression of PCNA and cyclins in the ASPP2^+/−^ mice was significantly higher compared with the ASPP2^+/+^ mice at 12 hours post-PH_X_, but rapamycin treatment attenuated or abolished the proliferative effect of ASPP2 deficiency (Fig. 5C,D). Furthermore, the number of PCNA-positive hepatocytes and the index of liver weight to body weight exhibited the same trend as PCNA expression in ASPP2^+/−^ and ASPP2^+/+^ mice post-PH_X_ (Fig. 5A,B). Taken together, these findings demonstrate that the inhibition of the mTORC1 pathway significantly suppressed liver regeneration in ASPP2^+/−^ mice, indicating that the enhanced liver regeneration observed in the ASPP2^+/−^ mice was dependent upon the mTORC1 pathway.

### Disruption of autophagic pathway further enhances liver regeneration in ASPP2^+/−^ mice

**Figure 6:**
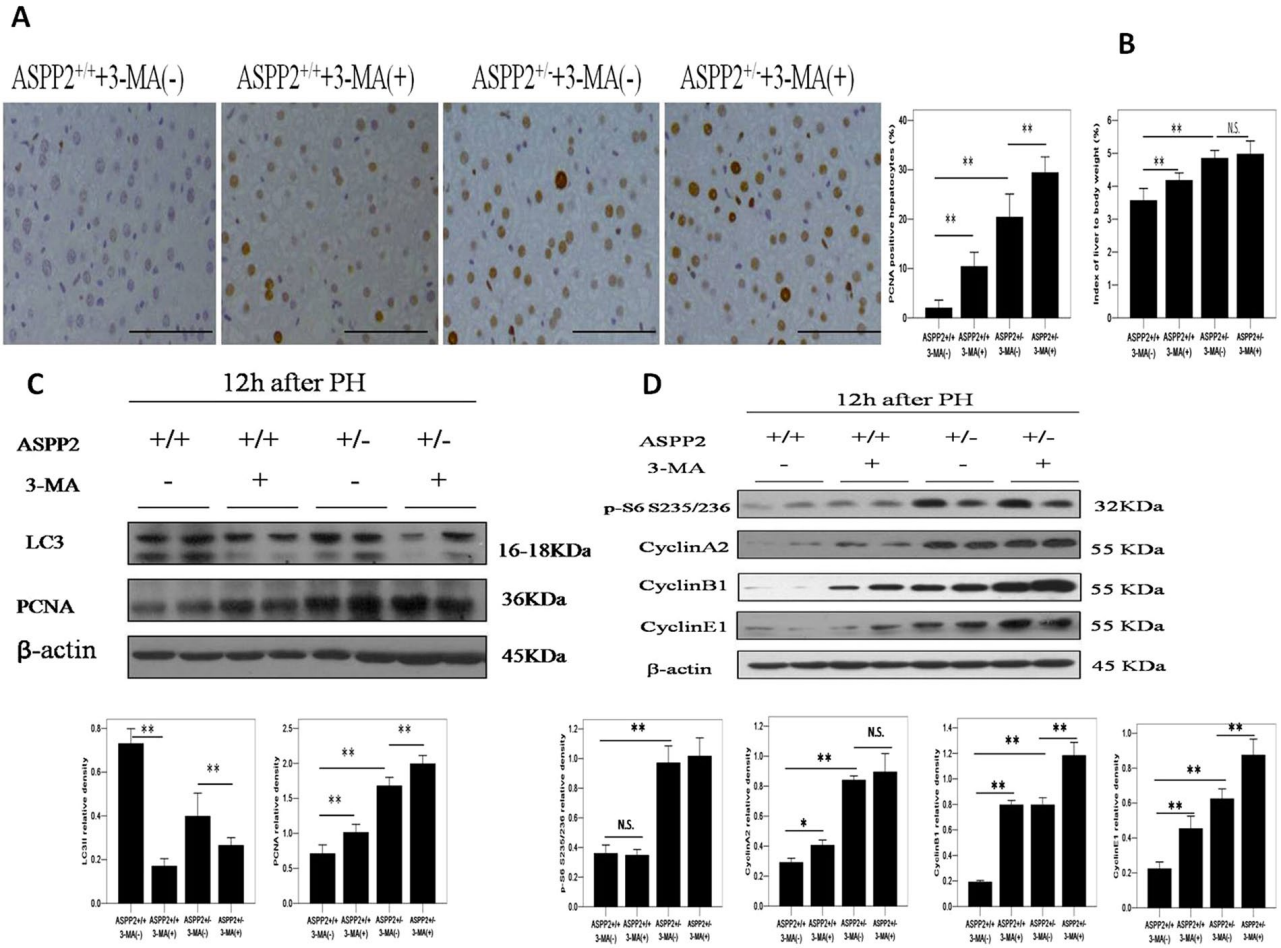
Disruption of the autophagic pathway further enhances liver regeneration in ASPP2^+/−^ mice. (**A**) Representative IHC staining of PCNA-positive hepatocytes in the livers of ASPP2^+/+^ and ASPP2^+/−^ mice treated with 3-MA or vehicle at 12 h after 70% PHx. Hepatocyte nuclei were counted in 10 microscopic vision fields per section. Three sections per mouse were examined (n = 5 for each time point). Scale bar: 100 μm. (**B**) Index of liver to body weight in ASPP2^+/+^ and ASPP2^+/−^ mice treated with 3-MA or vehicle at 24 h after 70% PHx (n = 5 for each time point). (**C**) Representative western blotting analysis of PCNA and LC3 expression in the livers of ASPP2^+/+^ and ASPP2^+/−^ mice treated with 3-MA or vehicle at 12 h after 70% PHx. (**D**) Representative western blotting analysis of phospho-S6 (Ser235/236), CyclinA2, CyclinB1 and CyclinE1 expression in the livers of ASPP2^+/+^ and ASPP2^+/−^ mice treated with 3-MA or vehicle at 12 h after 70% PHx. Quantifications were normalized to β-actin (n = 5 for each time point). Error bars represent the mean ± standard deviation (SD). *P < 0.05; *P < 0.01. Abbreviations: h, hour; N.S., no significant difference.

Considering that ASPP2 deficiency results in the inhibition of the autophagic pathway, we analyzed the role of the autophagic pathway in liver regeneration. 3-Methyladenine (3-MA) was used to block the autophagic pathway. As expected, there was no detectable LC3II conversion after 3-MA treatment in either the ASPP2^+/+^ or ASPP2^+/−^ mice (Fig. 6C). ASPP2 deficiency promoted the expression of PCNA and cyclinE1, which was significant higher after the 3-MA treatment compared with the vehicle treatment in the ASPP2^+/−^ mice at 12 hours post-PH_X_ (Fig. 6C,D). Furthermore, the number of PCNA-positive hepatocytes and the index of liver weight to body weight exhibited the same trend as PCNA expression in the ASPP2^+/−^ and ASPP2^+/+^ mice post-PH_X_ (Fig. 6A,B). Therefore, 3-MA treatment further enhanced liver regeneration in ASPP2^+/+^ or ASPP2^+/−^ mice post-PH_X_, indicating that the inhibition of the autophagic pathway promoted liver regeneration in mice post-PH_X_.

## Discussion

**Figure 7:**
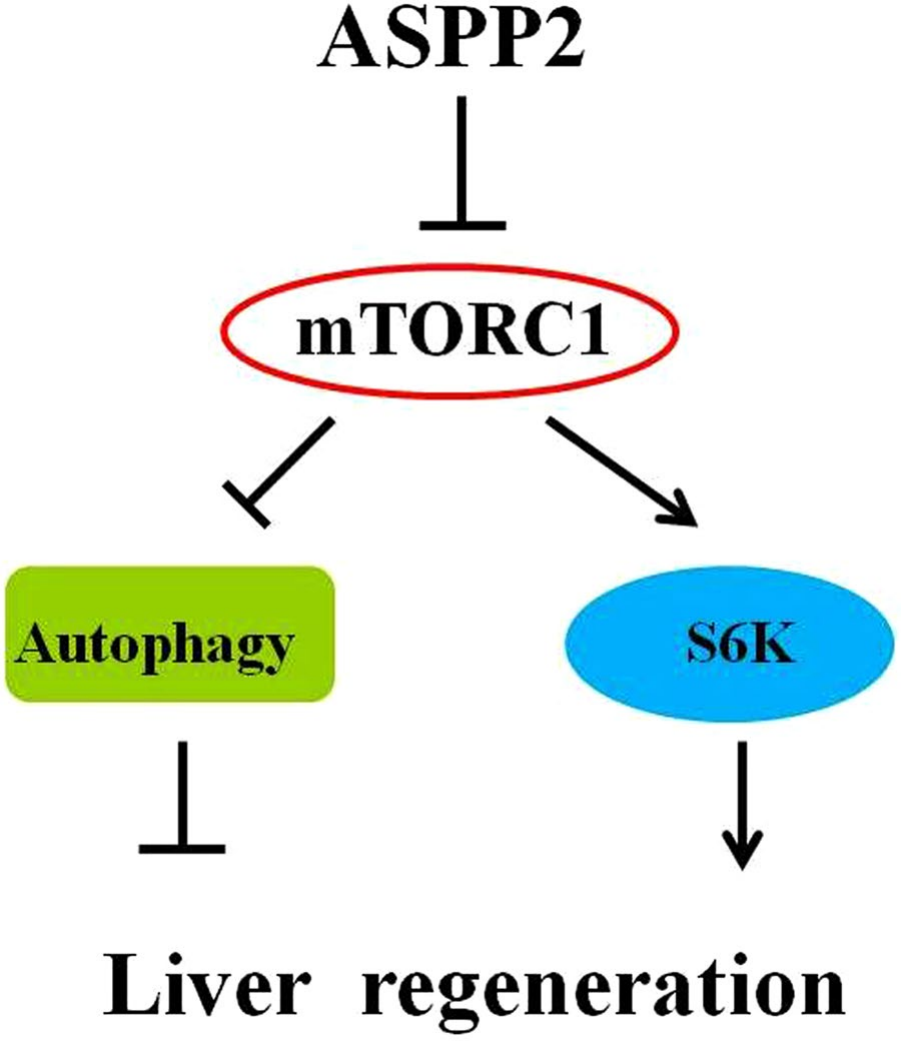
A model based on our studies, illustrating that ASPP2 inhibits liver regeneration through suppressing the mTORC1 pathway, which further regulates downstream molecules, such as p70S6K and those related to autophagy. In contrast, ASPP2 deficiency promotes liver regeneration through the activation of the mTORC1 pathway.

Although it is known that ASPP2 regulates cell apoptosis and growth, the role of ASPP2 in liver regeneration is still unknown. Here, we found that ASPP2 deficiency promoted liver regeneration through activating the mTORC1 pathway, which further regulated the expression of downstream molecules, such as p70S6K and those related to autophagy in the mouse model post-PH_X_ (Fig. 7).

The role of ASPP2 in liver regeneration has not been reported, but studies have shown that ASPP2 is involved in cell proliferation. Zhu *et al*. found that dioscin promoted osteoblast proliferation and differentiation by inhibiting cell autophagy via the ASPP2/NFκβ pathway, which indicated that ASPP2 was closely related to cell proliferation^13^. Liu *et al*. found that all-trans retinoic acid (ATPR) inhibited the proliferation of HepG2 cells by inducing G0/G1 cell cycle arrest and apoptosis through the upregulation of p53 and ASPP1 and the downregulation of iASPP, which indicated that the ASPP family may participate in cell cycle regulation^14^. In our study, the ASPP2 protein displayed a significant upregulation in wild-type (ASPP2^+/+^) mice at 12 hours post-PH_X_, and liver regeneration was enhanced in ASPP2 haploinsufficient (ASPP2^+/−^) mice at 12 hours after 70% PH_X_, which indicated that ASPP2 may play an inhibitory role in liver regeneration.

mTOR, which is a serine/threonine kinase that acts as a central regulator of important cellular functions, has two multiprotein complexes, namely, mTOR complex 1 (mTORC1) and mTOR complex 2 (mTORC2). mTORC1 regulates cellular growth, autophagy and apoptosis, and mTOR2 is involved in insulin sensitivity and cytoskeletal reorganization^15,16^. Therefore, the activation of mTORC1 is an important for stimulating cell growth and proliferation^17,18^. We found that the mTORC1 pathway was activated in ASPP2 haploinsufficient (ASPP2^+/−^) mice after 70% PH_X_. ASPP2 deficiency promoted liver regeneration, but the inhibition of the mTORC1 pathway reversed the proliferative effect of ASPP2 deficiency, which indicated that ASPP2 deficiency promoted liver regeneration through the activation of the mTORC1 pathway.

Our study found that ASPP2 can regulate the mTORC1 pathway in a mouse model with 70% PHx, but the underlying mechanism remains unknown. Our previous study determined that ASPP2 could bind to AKT in the nucleus and suppress the function of AKT^9^. mTORC1 is a major target of AKT, and mTORC1 is activated by AKT via phosphorylation; thus, we speculated that ASPP2 may regulate themTORC1 pathway through AKT. Another study demonstrated an interaction between p53 and mTORC1, and p53 is upstream of the repression of the mTOR pathway during ESC differentiation^19^. ASPP2 is a binding protein of p53, thus we speculated that ASPP2 may regulate the mTORC1 pathway through p53. Overall, we hypothesize that ASPP2 plays an important role in the mTORC1 pathway.

Some studies have indicated that there is a close relationship between autophagy and liver regeneration, but the results were controversial^20–22^. Toshima *et al*. found that liver regeneration and DNA synthesis are impaired in Atg5 knockout mice after PH_X_, indicating that the suppression of autophagy impaired liver regeneration^20^. Ni *et al*. found that hepatocyte proliferation was significantly increased in Atg5 liver-specific knockout mice with acetaminophen-induced liver injury, suggesting that the inhibition of autophagy promoted liver regeneration^22^. Our study showed that the inhibition of autophagy further enhanced liver regeneration, and the activation of autophagy suppressed liver regeneration in ASPP2^+/−^ mice post-PH_X_, indicating that autophagy may be a negative regulator of liver regeneration.

Previous studies showed that ASPP2 regulates autophagy^23–26^, but the mechanism was still unknown. Liu *et al*. showed that ASPP2 overexpression releases Beclin-1 from cytoplasmic Bcl-2–Beclin-1 complexes, and then, the released Beclin-1 induces autophagy in hepatoma cells, indicating that ASPP2 may induce autophagy^26^. Consistently, we found that autophagy was inhibited in ASPP2 haploinsufficient (ASPP2^+/−^) mice after 70% PH_X_, indicating that ASPP2 may promote autophagy. Considering that mTORC1 is one of the upstream regulators of autophagy^27–29^ and that ASPP2 regulates mTORC1, we speculated that ASPP2 may regulate autophagy through the mTORC1 pathway, which may be a novel finding that warrants further exploration.

Liver regeneration has critical clinical applications, including liver failure, cirrhosis and liver transplantation^30–32^. Our findings indicate that ASPP2 may be a negative regulator of liver regeneration, which may provide therapeutic targets for liver injury and hepatectomy in the future.

## Data Availability

The datasets generated during and/or analysed during the current study are available from the corresponding author on reasonable request.
